# Neurophenomenology of near-death experience memory in hypnotic recall: a within-subject EEG study

**DOI:** 10.1038/s41598-019-50601-6

**Published:** 2019-10-01

**Authors:** Charlotte Martial, Armand Mensen, Vanessa Charland-Verville, Audrey Vanhaudenhuyse, Daniel Rentmeister, Mohamed Ali Bahri, Héléna Cassol, Jérôme Englebert, Olivia Gosseries, Steven Laureys, Marie-Elisabeth Faymonville

**Affiliations:** 1 GIGA-Consciousness, University of Liège, Liège, Belgium; 2 Centre du Cerveau², University Hospital of Liège, Liège, Belgium; 3 GIGA-Sensation & Perception Research Group, University of Liège, Liège, Belgium; 4 Department of Algology, University Hospital of Liège, Liège, Belgium; 5 Department of Psychology, University of Liège, Liège, Belgium; 6 GIGA-Cyclotron Research Centre In Vivo Imaging, University of Liège, Liège, Belgium

**Keywords:** Consciousness, Perception

## Abstract

The neurobiological basis of near-death experiences (NDEs) is unknown, but a few studies attempted to investigate it by reproducing in laboratory settings phenomenological experiences that seem to closely resemble NDEs. So far, no study has induced NDE-like features via hypnotic modulation while simultaneously measuring changes in brain activity using high-density EEG. Five volunteers who previously had experienced a pleasant NDE were invited to re-experience the NDE memory and another pleasant autobiographical memory (dating to the same time period), in normal consciousness and with hypnosis. We compared the hypnosis-induced subjective experience with the one of the genuine experience memory. Continuous high-density EEG was recorded throughout. At a phenomenological level, we succeeded in recreating NDE-like features without any adverse effects. Absorption and dissociation levels were reported as higher during all hypnosis conditions as compared to normal consciousness conditions, suggesting that our hypnosis-based protocol increased the felt subjective experience in the recall of both memories. The recall of a NDE phenomenology was related to an increase of alpha activity in frontal and posterior regions. This study provides a proof-of-concept methodology for studying the phenomenon, enabling to prospectively explore the NDE-like features and associated EEG changes in controlled settings.

## Introduction

Many experiences, ranging from an altered perception of time to a sensation of separation from the body, may reflect cases of dissociative state as they involve mental separation of components (such as consciousness, memory, perception, and identity) that would ordinarily be processed together as an experience. Some of these non-ordinary states of consciousness seem closely related to the emergence of potential pathways for regulating awareness in crisis situations^1,2^. After having experienced situations of intense physical or emotional danger (e.g., severe trauma), some individuals may report dissociative states as well as vivid extra-ordinary and mystical perceptions, such as out-of-body experiences (OBEs), encountering with deceased relatives, or an intense feeling of peacefulness^3–5^. These phenomenological experiences are commonly referred to as “near-death experience” (NDE). It has been assumed that these subjective experiences are psychological responses to trauma in order to cope with it, which benefits the individual at that time^6^. A few decades ago, the prevalence of NDEs seemed difficult to apprehend. Most recently, studies have estimated their recall to be between 4–8% in the overall population^7–9^ and 10–23% when only considering cardiac arrest survivors^10–12^. Since the first descriptions of the phenomenon by Albert Heim^13^ and Victor Egger^14^, different sets of specific phenomenological features have been identified (e.g.^15–17^), ordered^18^, and quantified by standardized scales (e.g.^19^).

Recent research has led to the hypothesis that disruptions in information processing underlying dissociative detachment may be associated with reduced fronto-parietal synchronization^20–22^. Available scientific literature raises the hypothesis that dissociation may be related to decreased functional connectivity among brain areas, as measured by electroencephalography (EEG)^20,23,24^. Although dissociative states and their underlying mechanisms are attracting increasing interest due to significant theoretical and clinical implications, they are still poorly understood and have been under-studied in non-clinical samples. Hypnosis could be a great tool for producing modified states of consciousness that can yield dissociative states in order to contribute to a better understanding of the phenomenon^25–27^. Rooted in the psychodynamic tradition, hypnosis is characterized by a change in baseline mental activity through an induction procedure (notably reducing activity of the extrinsic brain network involved in the environment and sensory perception^28^). Hypnosis can be experienced subjectively as increased degrees of private processes, such as dissociation, absorption, reduced spontaneous thoughts and an altered perception of time^25,29–31^. Interestingly, hypnotic experience appears to create a ‘more real’ subjective experience and brain states closer to an actual experience, as well as facilitates focus on the recall of any kind of memory^25–27^.

In addition to its use in clinical contexts, hypnosis can be used in neuroscience research to study the neurobiological basis of hypnosis itself, but also as a tool to understand other phenomena^32^. The latter approach uses hypnosis in an instrumental manner to produce specific effects of interest and sometimes even models particular conditions^25,33,34^. Two previous studies suggested that NDE experiencers had safely re-experienced their NDE with a high level of multisensory awareness and in a very detailed way using hypnosis^35,36^. Using a 32-channel EEG, Palmieri and colleagues^26^ further suggested that associated electroencephalographic measures are suggestive of episodic memories of real events (notably because of the presence of theta activity associated with the recall of NDE memories) –although not necessarily corresponding to events in the external (real) physical world. Very recently, Facco and colleagues^37^ have succeeded in inducing OBEs under hypnosis in high hypnotizable people who neither experienced a NDE nor an OBE. EEG data captured by a 32-channel system showed a decrease in beta and gamma band activity in the right parieto-temporal area associated with a subjective OBE^37^. Overall, “recreating” NDE features in a controlled laboratory setting may allow to overcome the limitations inherent to the study of NDE (e.g., no monitoring of brain activity during the genuine NDE). Nevertheless, to date, no study has examined induced NDE phenomenology by hypnotic induction using high-density EEG in people who have already lived a ‘genuine’ NDE (i.e., NDE experiencers).

Given the unfeasibility to design a scientific study where subjects would experience a NDE in real-life situations and their unpredictability, we employed hypnosis to explore the NDE phenomenon through a within-subject comparison. To this end, we invited a group of individuals who had already experienced a genuine NDE to recall this event along with another emotionally positive autobiographical event dating to the same time period, in two conditions: during normal consciousness and during hypnosis. In addition, the present study has been designed specifically to prospectively and jointly explore the phenomenological experience using questionnaires and the associated neural correlates using high-density EEG. This method allowed to closely assess the experiencers’ subjective first-person phenomenological experience in parallel with state-of-the-art brain monitoring, combining the subjective experience and their neural correlates in a single setting. Given the very rich content information that characterizes NDEs^38–40^, we here focused on the two most frequently reported and characteristic features of the NDE experience during the recall: the intense feeling of peacefulness and OBEs^10,11,17,41^. Here, we show how to exploit hypnosis to provide first-person experience of a NDE phenomenology that simulates what experiencers had lived during their previous authentic experience.

## Results

### Behavioural results

No adverse event was observed in all our participants. Throughout the hypnosis sessions, participants evidenced slowed breathing and relaxed face.

In our sample of 5 participants, 3 were identified as highly hypnotizable subjects (see Table 1) according to the Stanford Hypnotic Susceptibility Scale (form C; SHSS:C^42^). The two other participants were identified respectively as a medium hypnotizable subject and a low hypnotizable subject.

**Table 1:** Participants’ Stanford Hypnotic Susceptibility Scale (form C; SHSS:C)^42^ total scores and responses to the Greyson NDE scale after the recall of the NDE memory in the two conditions.

Participants	SHSS:C score	Greyson NDE scale					
		Normal consciousness			Hypnosis		
		PE item	OBE items	Total score	PE item	OBE items	Total score
1	6	2	0	4	2	2	6
2	9	2	0	6	2	2	10
3	8	2	0	4	2	2	8
4	9	2	0	6	2	2	23
5	4	1	0	1	2	2	6

PE = feeling of peacefulness; OBE = out-of-body experience.

The pleasant autobiographical event (AUTOBIO; time since event: median = 28 years; range 11–58) happened within 5 years before or after the genuine NDE (time since NDE: median = 32 years; range 9–63). The time elapsed between the two events did not differ (Z = 1.62, p = 0.10, *r* = −0.51). Participants had Memory Characteristics Questionnaire (MCQ^43^) total scores that also did not differ significantly between the two memories (Z = 0.73, p = 0.46, *r* = −0.23; NDE: median total score = 90; range 72–94; AUTOBIO: median total score = 90; range 65–94), suggesting that both memories did not differ in terms of remembered phenomenological characteristics.

**Figure 1:**
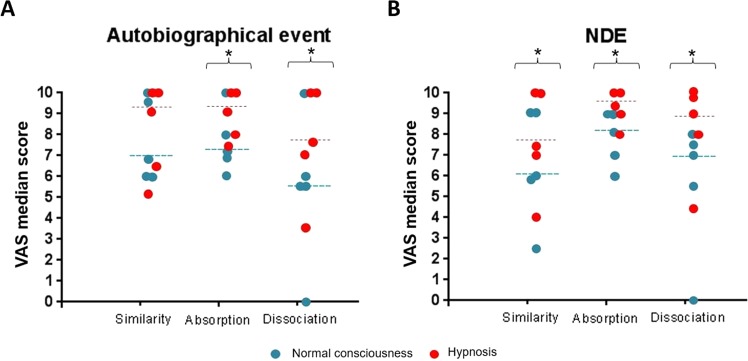
Participants’ Visual Analogic Scales (VAS) scores (and median, dashed lines) relating to level of similarity, absorption, and dissociation in normal consciousness (blue) and hypnotic state (red) for the autobiographical event (AUTOBIO) condition (**A**) and for the near-death experience (NDE) condition (**B**). *p < 0.05.

Figure 1 shows ratings of the level of similarity with the genuine event, absorption, and dissociation after normal consciousness (NC) and hypnosis (HY) conditions of each participant. For absorption and dissociation, participants reported significantly higher scores on the Visual Analogic Scales (VAS) in the HY condition than in the NC condition for both memories (for absorption: Z = 2.03, p = 0.042, *r* = −0.64; for dissociation: Z = 2.02, p = 0.043, *r* = −0.64). For the VAS related to the similarity with the genuine event, they reported significantly higher scores on the VAS in the HY condition than in the NC condition for the NDE memory (Z = 2.02, p = 0.043, *r* = −0.64). For the AUTOBIO memory, we did not observe significant difference regarding the similarity between the two conditions (Z = 0.94, p = 0.345, *r* = −0.30). Regarding the time estimation, one participant (highly hypnotizable) was unable to estimate the time elapsed for the two recalls under hypnosis. For NC conditions, participants estimated the time duration of the NDE recall to 12 ± 3 min, and of the AUTOBIO to 14 ± 4 min, as compared to 20 min (real time duration). For HY conditions, participants estimated longer time durations: for the NDE recall: 20 ± 7 min, and for the AUTOBIO: 18 ± 8 min, as compared to 20 min (real time duration).

When recalling the NDE memory in the NC condition, none of the participants’ reached the cut-off of 7 on the Greyson NDE scale (median total score = 4; range 4–6). All participants scored 0 (i.e., “not present”) on the Greyson NDE scale item assessing the presence of OBE. One participant scored 1 (i.e., “mildly or ambiguously present”) and the 4 other participants scored 2 (i.e., “definitively present”) on the Greyson NDE scale item assessing the feeling of peacefulness (PE). When recalling the NDE memory in the HY condition, the Greyson NDE total scores reached the cut-off of 7 for the 3 highly hypnotizable subjects (see Table 1), but the two other participants had a total score of 6/32. All participants scored 2 (i.e., “definitively present”) on the two Greyson scale items assessing the PE and the presence of OBE.

### EEG results

**Figure 2:**
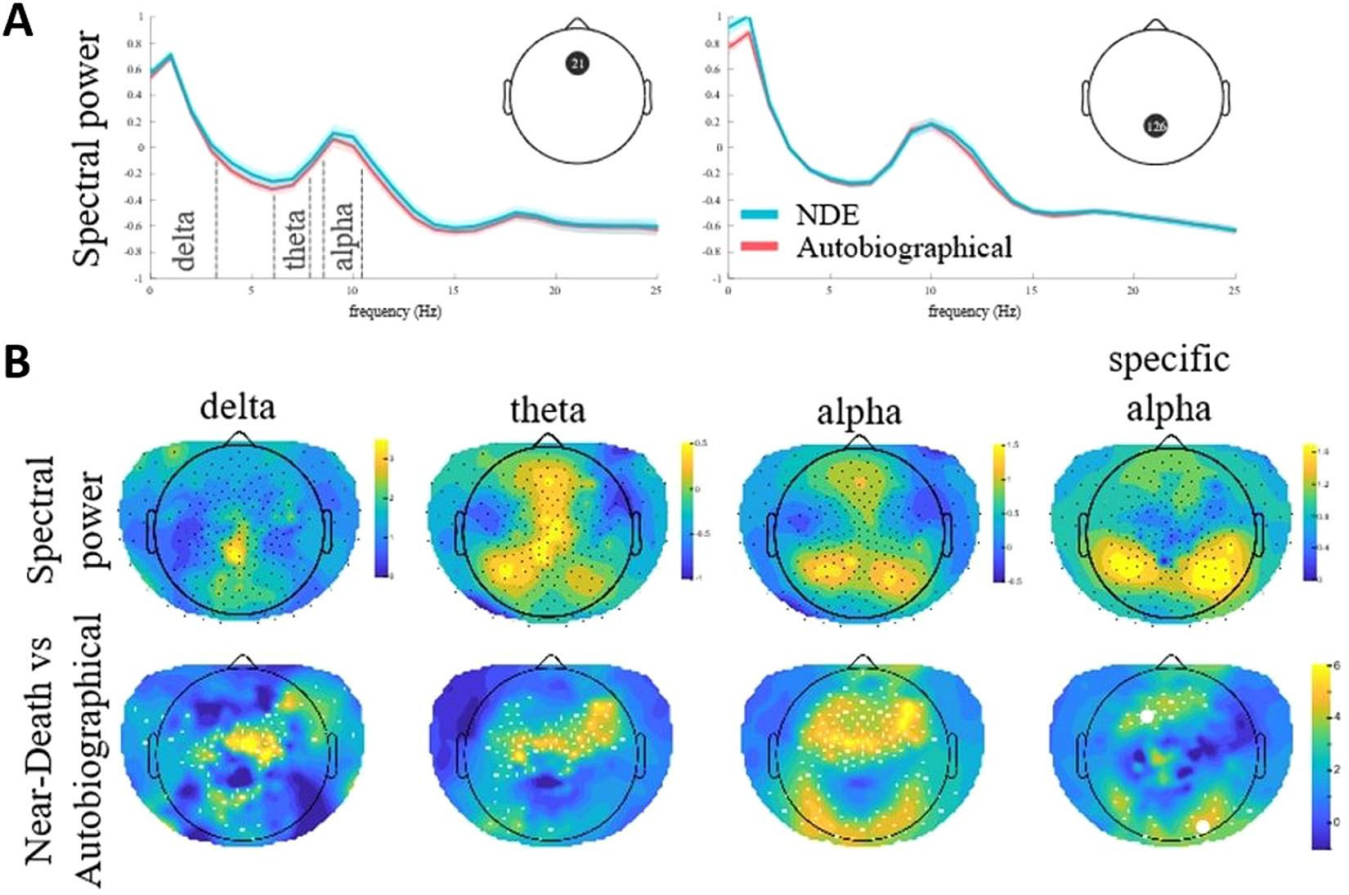
Effects of near-death experience (NDE) condition on spectral power (irrespective of whether this was in a hypnosis or normal consciousness experimental session). (**A**) The figure shows the power at a central frontal channel (left) and a central posterior channel (right), across all frequencies between 0 and 25 Hz. There are clear peaks at both channels at the low, delta frequencies, and around the alpha frequency which are then further examined. (**B**) The top panel shows the spectral power across all channels at selected frequency bands: delta (0.5–3.5 Hz), theta (7–8 Hz) and alpha (10–11 Hz) and “specific” alpha (alpha - theta band). The bottom panel shows the contrast between the EEG activity when participants described their NDE versus when they experienced another autobiographical event as T-values (i.e., mean difference normalized by variance). Regions in yellow indicate increased spectral power in the NDE condition (irrespective of whether this was in a hypnosis or normal consciousness experimental session). Significantly different channels are marked by white spots.

When exploring the topography (Fig. 2), we found that the NDE condition in NC and HY significantly increases the alpha power in both a frontal and posterior region at the scalp level. At a threshold of p-value < 0.016 these combine as a single cluster composed of 161 significant channels (peak channel = E2, right frontal; T = 7.298, p = 0.0009). The posterior region showed a significant alpha peak at channel E122 (left posterior; T = 5.486, p = 0.0014). To better examine possible interactions and covariates we focused on the two channels that showed peak NDE versus AUTOBIO differences above; E2 and E122. In the frontal electrode (E2), we found an interaction between HY_NC and NDE_AUTOBIO (χ2(1) = 8.318, p = 0.004) where NDE had higher alpha power during both HY and NC, it is significantly more so during NC. For the posterior peak electrode (E122), we found main effects for all HY_NC and NDE_AUTOBIO with no interactions between them. As in the frontal region, alpha power was higher during recall of the NDE (χ2(4) = 43.268, p < 0.001). Similarly, alpha power was significantly higher during hypnosis opposed to NC alone (χ2 = 62.297(4), p < 0.001). Interestingly, only for the posterior electrode we did find that alpha power significantly increases with SHSS scores (χ2(1) = 4.959, p = 0.026), while alpha tended to decrease with age (χ2(1) = 3.824, p = 0.051).

While the above analysis focuses on the condition effects in alpha power, as Fig. 2 illustrates, there may be substantial overlap in the effects from other bands. To isolate the specific effects on the alpha band in particular we examined the difference between the selected 10–11 Hz band and its lower neighbouring band (7–8 Hz). This analysis attenuated some of the effects, especially in more central electrodes, it highlighted a more frontal region as well as posterior areas, biased towards the right side. The most significant effect was an increase in alpha power during NDE (irrespective of whether this was in HY or NC) in a right posterior cluster of 49 electrodes (peak 167; T = 5.393, p = 0.008). The frontal cluster also showed an increase during NDE and consisted of 35 electrodes (peak 36; T = 4.982, p = 0.014).

**Figure 3:**
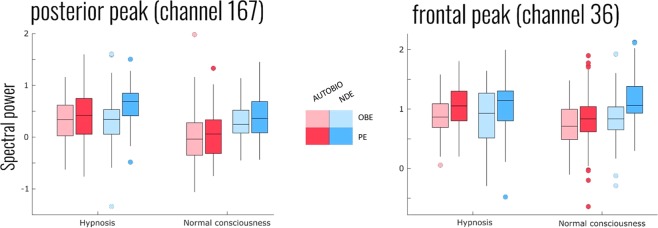
Spectral power of alpha activity for two selected channels with peak significant differences between near-death experience (NDE) and recollection of another autobiographical event (AUTOBIO) (highlighted in the bottom right of Fig. 3). The left graph shows the right-of-midline posterior channel also split by hypnosis (HY) and normal consciousness (NC) as well as recall of out-of-body experience (OBE)/kinaesthetic sensation (KS) (light coloured) or peacefulness (PE) (darker coloured). Here, the highest spectral power in NDE versus AUTOBIO is reflected in the NC condition, and only in the PE condition under hypnosis. The right graph shows the slightly left-of-centre frontal peak channel. At the frontal channel, the NDE condition has slightly higher spectral power during HY, but only for the PE condition during NC.

When the frontal peak channel (E36) was examined independently across all factors we found a significant interaction between HY_NC and NDE_AUTOBIO (χ2 = 9.593(1), p = 0.002) as well as a weaker interaction between NDE_AUTOBIO and OBE_PE (χ2 = 5.454(1), p = 0.019). This interaction is depicted in Fig. 3, and reflects a generally higher alpha power during hypnosis, NDE and PE conditions. Here both age (χ2 = 6.523(1), p = 0.011) and SHSS scores (χ2 = 15.917(1), p < 0.001) were also significant predictors of alpha power. We found no three-way interaction (χ2 = 2.183(1), p = 0.140) nor an interaction between HY_NC and OBE_PE (χ2 = 0.064(1), p = 0.801). For the posterior peak channel (E167), we again found a significant interaction between HY_NC and NDE_AUTOBIO (χ2 = 11.933(1), p < 0.001). As Fig. 3 shows, this interaction indicated that the increased alpha effect during NDE was only present in the NC condition (χ2 = 51.079(2), p < 0.001), and not during HY (χ2 = 0.372(2), p = 0.83). Also here we did not find a three-way interaction (χ2 = 0.550(1), p = 0.458) nor an interaction between HY_NC and OBE_PE (χ2 = 2.695(1), p = 0.101) nor an interaction between NDE_AUTOBIO and OBE_PE (χ2 = 1.711(1), p = 0.191). All results reported also show the same pattern when covariates of age and SHSS were not included in the model.

## Discussion

Our primary objective was to use a hypnosis-based protocol in the interests of prospectively exploring the brain activity underlying the NDE recall, as compared to the recall of another autobiographical event. We used a neurophenomenological approach to closely assess the experiencers’ subjective first-person phenomenological experiences in parallel with state-of-the-art brain monitoring (high-density EEG).

Based on participants’ self-reported responses, our hypnosis-based protocol increased the subjective experience in the recall of both memories, and led to a greater subjective ‘reality’ of the hypnotically induced experience as compared to the imagined one in NC, without any adverse effects. Indeed, at a phenomenological level, absorption and dissociation were reported as higher during HY conditions in all participants as compared to NC conditions, suggesting increased degrees of these processes. Participants reported significantly higher similarity with the genuine event in the HY condition for the NDE memory, but not for the autobiographical event memory. Understandably, these laboratory-induced experiences do not reflect initial NDEs and caution should be exercised in interpreting these results because the small sample size implies low statistical power. Nevertheless, the hypnosis protocol seems to have subjectively created phenomenological experiences that felt closer to the original NDEs.

When looking at the EEG, our results suggest that the NDE recall condition has measurable brain correlates. Mainly, our exploratory analysis showed that the NDE recalls were related to an increase of alpha activity in frontal and posterior regions. No other significant effects were found. Alpha-band activity has most often been associated with vigilance and attention and found in posterior areas^44,45^. As Fig. 2 shows, consistent with this literature, we found that alpha power is higher in bilateral posterior areas. However, the largest condition effects, especially those contrasting NDE with autobiographical memories are more fronto-central. Alpha activity may also reflect cognitive load on working memory. Increased alpha power was observed in both hypnosis conditions, but was noted only during the NDE recall in the case of the NC condition. It is therefore plausible that the NDE recall might trigger some sort of hypnotic-like state of dissociation even without the explicit induction of hypnosis. Based on the present results and our participants’ feedbacks, we hypothesize that the mere act of recalling a NDE (without an explicit induction of hypnosis) could lead to experience a spontaneous modified state of consciousness comparable to what can be lived during hypnosis. As in hypnosis, a subjective experience, through a modified state of consciousness spontaneously induced by the individual, may be spontaneously experienced from the encoding context when recalling a previously experienced NDE. It is now known that memory of critical situations (e.g., trauma) may be associated with powerful re-enactments of the event with intense sensory impressions^46^; however, the re-experiencing of the NDE memory has not yet been studied.

Interestingly, the level of alpha power in posterior regions, irrespective of actual hypnotic condition, was positively related to participant’s SHSS. However, this was not the case in the frontal area where the NDE effects were the strongest. This is not only indicative of the relationship between hypnosis and attention but also highlights the dissociation of the effect of the reproduced NDE-like features from attention and hypnosis alone. This frontal activity may then reflect the felt subjective experience in the recall of the NDE phenomenology induced by hypnotic induction. In addition, it does not seem likely that an increase of alpha activity in frontal regions would represent an enhanced relaxation during hypnotic induction in our participants. Indeed, a debate has arisen concerning the potential interpretation of alpha band in hypnotic state (e.g.^47^). Nevertheless, no relaxation measure was administered in participants in this study.

Our sample includes an unusually high proportion of individuals highly receptive to hypnotic state (i.e., 3 out of 5 participants), in view of the fact that subjects were enrolled in this study without knowing their hypnotisability a priori and without any selection criteria regarding the hypnotisability level. Interesting research questions for future research could be derived from this observation, such as knowing to what extent NDE experiencers may be conducive to hypnosis and to experiencing modified and altered states of consciousness. Some work has suggested an association between hypnotisability and the propensity to experience various anomalous phenomena^2,48^. We may assume that NDE experiencers have a propensity to enter dissociated states when faced with acute stress or other suitable physiological and/or psychological conditions. Hypnotic state can be regarded as a spectrum, covering distinct but related concepts such as dissociation or fantasy proneness. Very recently, we observed particularly strong engagement in fantasy in people who have recalled NDEs after non-life-threatening situations, and an association between the reported intensity of the experiences and this engagement in fantasy and imagination^49^. Greyson^50^ also observed NDE experiencers’ facilities to experience (non-pathological) dissociation states. While the retrospective and correlational design of their study limits the conclusions that can be drawn, further work should be carried out to characterize NDE experiencers’ cognitive profile and to observe to what extent their susceptibility (in any form whatsoever) could influence the availability of detachment and the appearance of phenomena such as NDEs. One may even hypothesize that a personality propensity toward dissociative experiences might be a factor predisposing people to experience NDE phenomenology. The idea that NDEs and hypnosis, although distinct phenomena, have probably some common processes has already been hypothetically mentioned (e.g.^51^). Nonetheless, empirical studies are needed to further investigate this question.

Using the short and long versions of the MCQ, several research teams recently assessed the NDE memory and the subjective experience associated with remembering this event^36,38–40^. They showed that the resulting NDE memory is very rich in details, containing even more phenomenological characteristics than any other experienced real event memories. Our results are however not consistent with these findings and rather showed that the NDE memory did not differ in terms of the amount of phenomenological characteristics as compared to the other autobiographical memory. Considering the long time elapsed since our participants experienced their NDE, this may be due to the fact that the other autobiographical memory recalled by themselves was inevitably a very salient memory which was strongly anchored in memory and would have benefited from a special encoding and storage (e.g., self-defining memory). A recent study highlighted the self-defining status of the NDE memory and demonstrated that NDE memories may constitute an important part of experiencers’ personal identity^52^. The two events recalled in this study were highly accessible and vivid personal memories that should probably correspond to self-defining memories.

At a methodological level, we chose to use a neurophenomenological approach^53,54^, in the sense that the subject was actively involved in the generation and the description of the subjective experience (first-person data) and that the EEG furnished “objective” brain electrical activity mapping (third-person data). As Varela^55^ initially proposed, both first-person and third-person data are linked and then can be analysed in a “mutual constraint” relationship. Furthermore, people vary in their ability to generate and report their first-person experience and these abilities can be enhanced through techniques, such as hypnosis or VAS respectively. This potentially helped the subjects to become aware of previously unavailable or inaccessible aspects of their experience. Thanks to this approach, we thus obtained phenomenologically enriched neurophysiological findings.

We assume that NDE-like memory, associated with positive emotions, may be induced in psychotherapy using hypnotic induction for therapeutic purposes. Moreover, a recent study showed that individuals who experienced embodiment using immersive virtual reality and witnessed a “simulated” NDE report positive life-attitude changes^56^. We thus believe that an integration of experimental and clinical research results might lead to the application of hypnosis in clinical and therapeutic contexts.

Finally, some limitations of this study should be acknowledged. Although promising, these preliminary results need to be interpreted with caution. Indeed, given the limited number of participants in the current study we are hesitant to over-interpret the increase of alpha activity in the NDE conditions. This result should be reproduced in a larger sample size before any firm links with other phenomena can be established. Another limitation is the fact that the NC and HY conditions were not randomized. The HY conditions were administered after the NC conditions for each subject since it is known that modified states of consciousness may have diverse aftereffects and that hypnosis can facilitate the recall of the memory^25–27^, leading to potentially recall details previously buried in the memory and therefore modify the subject’s memory. In addition, as hypnosis was used here as an instrumental manner to produce a NDE-like phenomenology, our results are even more difficult to interpret as current literature regarding hypnosis and associated brain changes seems dyshomogeneous^57^. Even though past research has shown that hypnosis leads to an objectively measurable brain change, results are mixed (e.g.^17,28,58,59^). Because the broad spectrum of activation pattern depends on both the hypnotic task itself and individual’s hypnotisability, this makes the interpretation of results difficult. Future studies are needed to better understand non-ordinary states of consciousness such as dissociative experiences or NDEs. However, this study represents a step in exposing the physiological underpinnings of a NDE phenomenology recall with hypnotic induction, and thus it adds to the emerging literature on the neural correlates of NDEs and hypnosis. Furthermore, this study outlines a controlled, within-subject experimental framework including the collection and accompanying analysis of EEG data, that is crucial for this potential future work.

In conclusion, we succeeded in recreating NDE-like features using hypnosis without any adverse effects in NDE experiencers. This preliminary study therefore demonstrates that a hypnosis-based protocol can be used for evoking a NDE phenomenology that closely resemble authentic NDEs and clearly indicates that there is value in exploring these avenues further. Our study suggests that the recall of NDE memories was related to an increase of alpha activity in frontal and posterior regions. This study provides a proof-of-concept methodology for studying the phenomenon of NDEs and presents promising results.

## Methods

### Participants

Participants were recruited via the GIGA-Consciousness and Centre du Cerveau² (University and University Hospital of Liège, Belgium). Five right-handed volunteers (mean age 57 ± 11 years; see Table 2) who reported having lived an experience that met the validated criteria of NDEs (i.e., Greyson NDE scale total score ≥7/32^19^) participated in the study after giving written informed consent. This study was approved by the Ethics Committee of the Faculty of Medicine of the University of Liège. The method was carried out in accordance with the Declaration of Helsinki. Exclusion criteria were: (1) psychiatric history; (2) neurological, cardiac, and respiratory diseases; (3) regular drug and alcohol consumption; and (4) signs of worry when suggesting to recall with hypnosis what they have experienced during the genuine NDE. One inclusion criteria was to have experienced an intense feeling of peacefulness/well-being and OBEs during the genuine NDE. None of the participants had previously experienced a hypnosis session.

**Table 2:** Participants’ descriptive and clinical data.

Participant	Age	Gender	Age at NDE	Greyson NDE scale total score	Circumstances
1	60	M	21	18	Traumatic injury (with loss of consciousness)
2	44	M	12	7	Tonsil surgery
3	66	M	57	15	Intense meditation state
4	71	F	8	15	Surgery (removal of a foreign object)
5	48	F	18	12	Traumatic injury (with loss of consciousness)

### Procedure

Prior to the day of the experimental sessions, each participant was seen by a certified clinical psychologist (D.R.) for an in-depth clinical interview to detect any psychiatric, neurological, personality or psychological disorder and, more specifically, to screen for any acute psychiatric decompensation, notably using the Structured Clinical Interview for Diagnostic and Statistical Manual of Mental Disorders (DSM-IV-Tr^60^).

**Figure 4:**
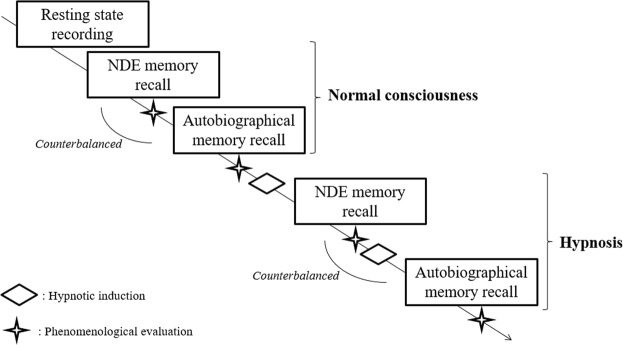
Procedure of the experimental session.

During the experimental session, participants were asked to recall their NDE and one of their most emotionally positive salient (real) autobiographical memories (control condition). Detailed information about these two events was obtained through a semi-structured interview as described elsewhere (see^61^). This semi-structured interview and the hypnotic induction were conducted by an experienced professional anaesthesiologist well-credentialed in the use of hypnosis (M.E.F.). Phenomenological characteristics of these two memories were assessed using a short version^62^ of the Memory Characteristics Questionnaire (MCQ^43^). Then, the participant underwent a high-density EEG recording session using a cap containing 257-channel (to a Cz) electrodes (Electrical Geodesic Inc. NA 300 amplifier; sampling rate of 250 Hz). Impedances were kept below 50KΩ whenever possible. The procedure of the session is outlined in Fig. 4. The participant was comfortably seated and a resting state recording was first performed for 5 min with eyes closed (as participants lie with the eyes closed for the normal consciousness and hypnosis conditions) to provide a baseline measure of brain activity. The participant was then instructed to perform two tasks in controlled conditions of mental imagery (without any hypnotic induction): imagine their NDE and their pleasant autobiographical event for 20 min each (*normal consciousness* (NC) *conditions*). The order was counterbalanced between participants. Following this, a hypnotic state was induced in the participant in the same way as in our patients during surgery (e.g.^63–65^) and as in our previous functional neuroimaging studies with healthy volunteers (e.g.^29,61,66^). The hypnotic instruction included a 5-min induction procedure with eye fixation (ultimately closing the eyes) and progressive muscle relaxation. Permissive and indirect suggestions were used to develop and deepen the hypnotic state. The participant was then invited to re-experience their NDE memory (20 min) and their pleasant autobiographical memory (20 min) in a counterbalanced order, which were the *hypnosis* (HY) *conditions*. M.E.F. used the personal written narratives and the words stated by the participant in the semi-structured interview to help them recalling the memories. The participant was continuously given cues for maintaining a hypnotic state. The exact words of the induction technique and specific suggestions and details during the course of the induction varied depending on the hypnotist’s (M.E.F.) observation of participant behaviour, and on her judgment of participants’ needs (as in her previous clinical experience with hypnosedation; see^67^). For each NDE memory recall, the session was separated into two phases: focusing on the intense *feeling of peacefulness* (PE) and the *out-of-body experience* (OBE) experienced during the genuine NDE (±half of the recall time; guided by M.E.F.). Regarding the other autobiographical memory, each recall was separated into two phases: focusing on the *feeling of peacefulness* (PE) and the *kinaesthetic sensations* (KS) experienced during the genuine event (±half of the recall time; guided by M.E.F.).

After each event recall, the participant was invited to relax and to spontaneous tell what he/she felt and experienced. The participant was then invited to use a self-scoring system of hypnotisability experience (i.e., rating the level of similarity with the genuine event, absorption and dissociation)^68^. The participant was also asked to estimate the time elapsed during each recall. Finally, the Greyson NDE scale^19^ was administered.

After the experiment session, on a separate day, hypnotisability was assessed with a French version of the Stanford Hypnotic Susceptibility Scale (form C; SHSS:C)^42^. A phone call was made by the clinical psychologist three days after the EEG session to be sure that the participant was fine. This gave us the opportunity to observe any physical or psychological aftereffects of the hypnosis session.

### Self-report questionnaires

#### Memory characteristics questionnaire (MCQ)

The short version^62^ of MCQ^43^ includes 16 rating items assessing feeling of re-experiencing, visual details, other sensory details, location, time, coherence, verbal component, emotion while remembering, the belief that the event is real, one’s own actions, words and thoughts, visual perspective, emotional valence, personal importance, and reactivation frequency. Participants were requested to rate each item using a 7-point Likert scale and a total score was derived summing all the 16 items referring to as the amount of memory characteristics (i.e., higher total scores reflect greater amount of memory characteristics).

#### Greyson NDE scale

This scale is a validated multiple-choice tool used to permit a standardized identification of NDEs with a total cut-off score of 7 (out of 32)^19^. It assesses 16 NDE core content features, including the feeling of peacefulness and OBE. For each item, the scores are arranged on an ordinal scale ranging from 0 to 2 (i.e., 0 = “not present”, 1 = “mildly or ambiguously present”, and 2 = “definitively present”).

#### Self-scoring system of hypnotisability experience

Participants rated their subjective experiences concerning the level of similarity with the genuine event, as well as their level of absorption and dissociation, using VAS. This measuring instrument enables to quantify subjective characteristics or attitudes that cannot be easily directly measured^69^ and are believed to range across a continuum. The VAS were presented as a 100-mm rule with a movable cursor. Scores were derived by measurement in centimetres from the left of each scale where “not at all” corresponded to 0 and “fully” to 10.

#### Stanford hypnotic susceptibility scale

Hypnotic susceptibility was measured with the Stanford Hypnotic Susceptibility Scale (form C; SHSS:C)^42^. The SHSS:C is currently considered as the gold standard for hypnotic susceptibility testing^70^. According to this scale, individuals obtaining scores ≤ 4 are defined as low hypnotizable; scores between 5 and 7 are defined as medium hypnotizable; and scores ≥8 are defined as highly hypnotizable. We used a French version of the scale.

### EEG preprocessing

Data were then imported into Matlab (The MathWorks, Inc., Natick, Massachusetts, United States.), and further processed using custom, open-source analysis scripts (https://github.com/CSC-UW/csc-eeg-tools). Each step was performed blind to the start or end of any particular session and to the session order. A first-order high pass filter was applied at 0.1 Hz, followed by a second-order bandpass butterworth filter from 48 to 52 Hz to attenuate 50 Hz mains power noise. All non-session EEG data, identified using manual triggers during the recording, was then removed as this was mainly filled with movement artefacts. The remaining data were then visually examined and manually marked for bad channels (4, 11, 24, 35, 64 channels were bad for each of the participants) and bad portions of data (1.0%, 3.2%, 3.4%, 6.6%, 10.4% artefacts). Independent component analysis was then run only on the remaining data using the first 60 principal components to ensure independence of the time series input and that sufficient data points were available given the number of independent components to be estimated^71^. Components were manually rejected (17, 21, 26, 30, 35 components removed for each of the participants) after inspection of their respective time series, projected topography, spectral power, and a manual inspection of the effect of their removal on the channel time series. Bad channels were then re-interpolated using spline interpolation. All channels were re-referenced to the average reference^72^, recuperating the central reference channel resulting in a clean recording of 257 channels over approximately 40 min for each participant.

For spectral analysis, the data was segmented into 30 sec, non-overlapping epochs. This length was chosen rather arbitrarily with the constraints that sufficient data should be included to obtain a stable estimate of the spectral power within that segment, but resulting in a sufficient number of segments to use in the linear mixed model. Spectral power within each of these epochs was estimated using the p-welch algorithm (windows of 1 sec duration, with a hanning window and 50% overlap; https://www.mathworks.com/help/signal/ref/pwelch.html). Particular parameters of the algorithm were chosen with emphasis on reducing the effects of nonstationarity on the spectral estimate while maintaining a sufficient frequency resolution (1 Hz bins) for relatively high precision in reporting pf effects.

### Statistical analysis

#### Self-report questionnaires

Given that our study included a small sample size, nonparametric tests were used to compare rating responses within the different recall conditions. We performed Wilcoxon signed-rank tests to compare the elapsed time between the two events, MCQ total scores, and ratings regarding the level of similarity with the genuine event, absorption, and dissociation.

#### EEG

Statistical analysis was performed for all channels over selected frequency ranges. These ranges were chosen by examining the spectral pattern over the frontal, central and posterior, midline channels for those frequencies which stood out over the expected 1/f pattern (see Fig. 2). This was done over the combination of all conditions and over all participants and 3 ranges were found: a low delta range of 0.5–3.5 Hz, an alpha range from 9.5–11.5 Hz, and a low-beta band of 16.5–19.5 Hz. The single dependent variable of spectral power at these ranges were calculated by taking the square root of the mean of the log power values squared for each 30 sec window. Any window with more than 5 sec of marked artefact was rejected from further analysis. This left 623 windows over the 6 conditions and 5 participants for statistical analysis.

Linear mixed models were used to model the data with the participant identity as a random variable in order to account for the non-independence of each window coming from a single participant. Using the values from each 30 sec window for each participant (as opposed to the mean spectral power for each condition), not only allows us to have a better estimate of the stability of the power calculation within each participant, but it also takes into consideration how much data comes from each participant. This is crucial in our setting as the borders of the OBE and PE conditions were set slightly differently by the experimenter and some participants only completed a few minutes of some particular condition and would thus be weighted accordingly. By taking the spectral estimates of the alpha band at the central channel, distinct models were created and compared using the Akaike’s Information Criteria until the simplest, but significantly lowest, model was found. The central Cz channel was chosen a-priori to test relevant models since it is unfeasible to test for the optimal models at each electrode individually. The best model was the three-way interaction (and all lower-order effects) between the experimental conditions HY versus NC (HY*_*NC), *NDE* versus *autobiographical* (NDE_AUTOBIO) and OBE versus PE (OBE_PE). Along with this interaction, the main effects of the covariates of participant age, and their SHSS scores were also found to contribute to the best model.

For the full topographic analysis of all channels we repeated this linear mixed model calculation over all of the 257 channels independently using the same mixed model. However, applying the same test for each channel and at each selected frequency band presents a serious multiple comparisons issue. We therefore implemented a non-parametric permutation approach combined with threshold-free cluster enhancement (TFCE) to increase both the specificity and sensitivity^73–75^. The TFCE approach examines the observed T-values of each model parameter and each channel, and adjusts them based on the support from their neighbouring channels. These TFCE values are determined to be significant using the classic “maximum permutation approach”, which relies of the creation of an empirical null distribution of results to which the original values are compared to. The empirical distribution is created by randomizing the labelling of the NDE_AUTOBIO factor in the original data. For each randomized dataset, only the maximum value over all channels is taken to be a part of the empirical distribution for that model parameter. The p-value is then determined by calculating the percentage of these maximum randomized values which shows greater effect sizes (i.e., TFCE values) than the original labelings for each channel. Two thousand two hundred randomized datasets were calculated to form the empirical distribution. Given that this entire procedure was applied over the 3 selected frequency ranges we controlled for this element of multiple comparisons by reducing our alpha threshold by a factor of 3 for significance cut-off of 0.016. While reporting a standardized effect size may also be helpful to understand the data, using the mixed model approach, combined with the TFCE correction of the data, no such measure is currently available.
